# Psychometric properties of the Confidence and Trust in Delivery Questionnaire (CTDQ): a pilot study

**DOI:** 10.1186/1472-6874-12-26

**Published:** 2012-09-01

**Authors:** Elke Jeschke, Thomas Ostermann, Natalie Dippong, Dagmar Brauer, Harald Matthes

**Affiliations:** 1 Havelhoehe Research Institute, Kladower Damm 221, 14089, Berlin, Germany; 2 Center of Integrative Medicine, University of Witten/Herdecke, Gerhard-Kienle-Weg 4, 58313, Herdecke, Germany

**Keywords:** Validation study, Delivery, Questionnaire, Trust, Confidence

## Abstract

**Background:**

Assessing expecting mother’s opinions prior to birth draws a comprehensive picture for the caregivers about their emotional state and their expectations. Some questionnaires to cover these aspects do exist. This study aims to present the psychometric properties of a new instrument, the Confidence and Trust in Delivery Questionnaire (CDTQ) a short but reliable a self-report instrument that focuses on confidence and trust as meaningful dimensions for expectant mothers.

**Methods:**

A pilot validation study of 221 women 6 weeks before childbirth was conducted in Germany between October 2007 and June 2008. To detect structural relations between the items, factor and reliability analyses were applied to the CTDQ items. Factor analysis was performed by means of principal components analysis and varimax rotation. Internal reliability was assessed by Cronbach’s alpha. External validation was performed using the sense of coherence (SOC) scale.

**Results:**

The CTDQ comprises of 11 items. We found a 4-factor structure. The internal consistency of the whole item pool (Cronbach’s α = 0.79) and the 4 subscales [confidence in labor (α = 0.82); partner’s support (α = 0.62); trust in medical competency (α = 0.68); being informed (α = 0.60)] can be regarded as sufficient or even excellent. The 4 factors explained 69.6% of total variance. Except for a high intercorrelation (0.70) between “partner’s support” and “trust in medical competence”, the subscales show low intercorrelations, indicating an adequate independence of the respective subscales. Regarding the external validity we found minor respective moderate correlations with the SOC scale.

**Conclusions:**

Our data suggest that the CTDQ is a useful instrument to assess confidence and trust in delivery. With 4 clinically relevant dimensions, the CTDQ is now open for further studies in the field of labor.

## Background

The expectation of childbirth is a fundamental experience in a woman’s life. There is no doubt that pregnant women fear the intense pain of giving birth and the feeling that something might go wrong. Expectations do vary among women with respect to her social status, educational level, previous experience with childbirth and the level of available information on childbearing [1-4]. Independent of those dimensions, both qualitative research and quantitative evaluations have identified a variety of factors, which seem to have important meaning for expectant mothers. According to a literature review by Hodnett in 2002 [5], trust, confidence, hope and the feeling of being involved in the decision-making process seem to be so important that they suppress other factors including demographic aspects like age, socioeconomic status, ethnicity, childbirth preparation, environment, pain and medical interventions in their importance to mothers-to-be. Having trust and confidence in labour is critical to women’s perceptions of care and may influence their choices and decisions as well as feeling about themselves. Assessing women’s opinions prior to birth therefore draws a comprehensive picture for the caregivers about their emotional state and their expectations than surveying the mothers afterwards. Already in 1987 Corbin i.e. found that a perceived sense of control of pregnant women led to higher levels of confidence and trust in their own and the health team's management efforts [6]. Thus questionnaires may be used to detect early problems of upcoming mothers with themselves or the setting which may result in improved childbirth outcome.

There are some instruments that have already addressed the expectations of childbirth in a reasonable and valid way. The Childbirth Expectations Questionnaire (CEQ) [7] developed by Gupton in 1991, for instance, is a 35-item instrument including the dimensions “cope with pain”, “nursing support”, “partner’s support” and “medical interventions”. The Wijma Delivery Expectancy/Experience Questionnaire (W-DEQ) [8] introduced by Wijma in 1998 is mainly related to the aspects of fear, lack of positive anticipation and riskiness in childbirth, while the Childbirth Self-Efficacy Inventory (CBSEI) [9] introduced by Lowe in 1993 focuses on self-efficacy and coping expectancies. Finally, the Cambridge Worry Scale (CWS) was developed to assess the content and extent of maternal worries in pregnancy, which since 2009 is the only one with a validated German version [10].

However, we wanted to develop a short but reliable instrument that focuses more on the fundamental aspects of confidence and trust as meaningful dimensions and sources of support for expectant mothers. To our knowledge, no validated German instrument has been developed to cover these aspects sufficiently.

Thus, this pilot survey aims to create and evaluate a practice-oriented, short but reliable instrument and to examine its statistical properties in a survey of expectant mothers 6 weeks before childbirth.

## Methods

### Questionnaire development

An item pool of 15 items was first generated through the use of literature reviews and interviews of midwives and expectant mothers. After the removal of similar items, 13 were considered suitable for the questionnaire pool. An expert panel, consisting of 4 midwives, 2 physicians and 2 methodologists (one statistician and one epidemiologist), discussed these items both from the point of content validity but also from the viewpoint of construct coherence. After one discussion round and one rephrasing session, a final 11-item questionnaire was tested for compliance in a small sample of mothers and then consented and approved for the pilot study.

### Study setting and participants

The pilot validation study was conducted in Germany in the maternity ward of a general hospital with a specialization in integrative medicine between October 2007 and June 2008. Six weeks before childbirth, 318 women who attended the maternity ward for booking were consecutively invited by their midwive to participate in the study. Mothers were approached while waiting in the waiting room. They were informed that completion of the questionnaire was voluntary and were assured confidentiality in a comprehensive counseling session given by the midwive. They were also informed that the purpose of the study was to better understand the attitudes and beliefs of upcoming mothers. Women who did not give written informed consent were excluded. Due to internal quality management reasons, women who delivered in another hospital were also excluded.

Of the 318 potential participants, 38 (11.9%) declined, 29 (9.1%) insufficiently completed the questionnaires and 30 (9.4%) decided to give birth elsewhere. The final sample included a total of 221 from 318 women (69.5%).

### Ethical considerations

As this was a non invasive epidemiological study there was no necessity to obtain a vote from a local research ethics comitee [11]. However the rules for good epidemiological practice were fully applied.

### Data collection

The study included three questionnaires.

1. The Confidence and Trust in Delivery Questionnaire (CTDQ) consists of 11 7-point Likert-scaled items ranging from 1 (“very”) to 7 (“not at all”) that ask questions related to confidence, support and actual information about labor and epidural analgesia (EA) see Table 1 for the questionnaire items and Additional file 1 for the German translation). The CTDQ was developed by methodologists, physicians and experienced midwives who work in the maternity ward.

**Table 1:** CTDQ: Factorial structure and item reliability (0.789)

**Item**	**Missing values**	**Mean**	**SD**	**Item-diff. index**	**Factor loading**	**Cronbach’s α**		**r**_**Item-total**_	
						**Total**	***scale-wise***	**Total**	***scale-wise***
**Confidence in labor**							**0.824**		
Upcoming labor is a source of joy	2	2.42	1.35	0.35	**0.848**	0.755	0.746	0.577	0.717
When I think of labor and its pain I tend to eagerly anticipate it	-	3.15	1.39	0.45	**0.825**	0.763	0.775	0.520	0.655
Narratives and reports of labor have strengthened my joy and confidence	-	3.40	1.35	0.49	**0.750**	0.748	0.785	0.632	0.633
When I think of labor I am sure to cope with the pain	-	2.86	1.33	0.41	**0.732**	0.764	0.804	0.512	0.589
**Partner’s support**							**0.619**		
It is very important to me that my partner is at my side during labor	5	1.33	0.83	0.19	**0.822**	0.792	0.606	0.213	0.365
During labor there will always be a person I can trust	-	1.51	0.95	0.22	**0.727**	0.767	0.468	0.511	0.464
I can trust the support of my partner during labor	3	1.45	0.82	0.21	**0.614**	0.777	0.474	0.418	0.464
**Trust in medical competence**							**0.675**		
I can trust the obstetrician during labor	3	2.13	1.14	0.30	**0.885**	0.786	-	0.312	0.560
I can trust the midwife during labor	6	1.58	0.78	0.23	**0.824**	0.776	-	0.426	0.560
**Feeling informed**							**0.595**		
I feel well informed about epidural analgesia	-	2.67	1.50	0.38	**0.921**	0.802	-	0.242	0.444
I feel well prepared for my labor	2	2.58	1.14	0.37	**0.664**	0.755	-	0.599	0.444

2. A self-report questionnaire asking for socio-medical and demographic background information about education, social status, age, and the use of supportive measures.

3. The “Sense of Coherence” scale (SOC) [12] consists of 29 items. Every item is rated on a 7-point likert-scale giving a maximum score of 203 with higher scores indicating a good sense of coherence. The SOC can be divided in three components: “Comprehensibility” (11 items, e.g. “Do you have very mixed-up feelings and ideas?”, Range 11–77 ), “Manageability” (10 items, e.g. “Do you have the feeling that you’re being treated unfairly?” , Range 10–70), and “Meaningfulness” (8 items, e.g. “How often do you have the feeling that there’s little meaning in the things you do in your daily life?”, Range 8–56). According to recent findings in obstetrics, a high SOC helps women cope with pain during delivery. The study by Sjostrom et al. showed that the SOC scale measures the capacity to cope with the unforeseeable circumstances that child-bearing still involves today [13]. Oz et al. demonstrated that a high SOC was an independent predictor of an uncomplicated delivery [14]. Therefore we decided to use the SOC and its subscales as indicators for external validity.

Women completed the questionnaires (self-report questionnaire, CTDQ, SOC) between the 34th and 40th week of their pregnancy and returned the questionnaires to their midwife in a closed envelope before labor started. For completion of the questionnaires space from the waiting room of the ward was seperated in order to guarantee privacy after having received the women’s consent. Questionnaires then were dropped into a box, collected entered into a database by an independent documentation officer using a automated scanning process to guarantee anonymity of the participants.

### Statistical analysis

To detect structural relations between the items, factor and reliability analyses were applied to the CTDQ items. Factor analysis was performed by means of principal components analysis and varimax rotation in order to arrive at the solution that demonstrates both the best simple structure and the most coherence. Tests on sampling adequacy (Kaiser-Meyer-Olkin criterion) and multicollinearity (Bartlett test of sphericity) were undertaken prior to factor extraction to ensure that the scale items were appropriate for principle component analysis. A Kaiser-Meyer-Olkin criterion ≥ 0.50 and a Bartlett test of sphericity with p < 0.05 were regarded as mandatory for factor analysis [15]. Examination of the internal consistency of the item pool was performed by calculating the Cronbach’s alpha coefficient for both the complete item pool and the subscales generated by the factor analysis. Meaningful factors were retained and interpreted on the basis of their psychometric properties. Additionally, internal reliability was further assessed by means of item-total correlations and a calculation of the item-difficulty index.

Finally, external validation of these factors was performed by correlation of the subscales with the subscales of the SOC questionnaire [12,16] taking into account the results of Sjöström et al. [13], who found that women’s SOC is an indicator of their capacity to cope with the process of childbearing. To obtain information on the independence of the CTDQ and the SOC the proportion of shared variance between the subscales was calculated by means of r^2^, the square of the correlation coefficient. Statistical analysis was performed using SPSS 18.0 for Windows.

## Results

### Study population

The mean age of participants was 31.9 years (SD 4.9; range 19–45). 177 (80.1%) of them attended a childbirth preparation class and 125 (56.6%) were expecting a child for the first time. Complete demographic and socio-medical information on all participants is provided in Table 2.

**Table 2:** Demographic and socio-medical information of participants

**Participants**	
**Total[N (%)]**	**221 (100)**
Sociodemographic factors	
Age in years [Mean ± SD]	31.9 ± 4.9
Higher education* [N (%)]	197 (89.1)
Attendance at a childbirth preparation class [N (%)]	177 (80.1)
Parity [N (%)]	
1	125 (56.6)
2	53 (24.0)
3	11 (5.0)
4	4 (1.8)
Missing values	28 (12.7)

* having more than 10 years of schooling.

### Dimensions and internal reliability of CTDQ

We found a 4-factor model with a Kaiser-Meyer-Olkin measure of sampling adequacy of 0.789 and a highly significant Bartlett test of sphericity (p < 0.001). The cumulative variance explained by this model was 69.6%. The internal consistency of the whole item pool (Cronbach's α = 0.789) and the respective subscales (Cronbach's α between 0.595 and 0.824) can be regarded as sufficient or even excellent. Both factor structure and reliability parameters are shown in Table 1.

The first factor with 4 items describes “confidence in labor (CL)” with items such as “upcoming labor is a source of joy”. Its internal consistency is excellent (Cronbach's α =0.824); item-difficulty values between 0.35 and 0.49 can be regarded as excellent (Table 1). Factor 2 consists of 3 items dealing with “partner’s support (PS)”. Internal consistency (Cronbach's α =0.619) was sufficient, while the item-difficulty (between 0.19 and 0.22) indicates strong floor effects. The next factor with 2 items shortly describes “trust in medical competency (TMC)” with a reliability of α =0.675. Item-difficulty again was quite low, ranging from 0.23 to 0.30. The last factor describes the feeling of “being informed (BI)” about labor and is comprised of one general item (“I feel well prepared for my labor”) and one special item on EA (“I feel well informed about EA”). This factor shows the lowest reliability (Cronbach's α =0.595) with an acceptable item-difficulty of around 0.37.

As seen in Table 3, only the “partner’s support” correlated strongly with “trust in medical competence” (r = 0.702) while the other subscales showed moderate correlations between r = 0.184 and r = 0.334. With values between 0.213 and 0.632 for the complete instrument and values between 0.365 and 0.717 for the subscales, item-total correlations are in the optimal range for psychological test instruments [17].

**Table 3:** Correlation of CTDQ -Scales with SOC-29 scale and pain level

		**CTDQ**				**SOC**			
		**Confidence in labor**	**Partner’s support**	**Trust in medical competence**	**Feeling informed**	**Total**	**Comprehensibility **	**Manageability **	**Meaningfulness**
**CTDQ**	** Confidence in labor**	1	0.278**	0,184**	0,334**	−0,305**	−0.187**	−0.325**	**−0.294**
	**Partner’s support**		1	0.702**	0.254**	−0.274**	−0.180**	−0.321**	−0.201**
	**Trust in medical competence**			1	0.223**	−0.212**	−0.158*	−0.238**	−0.142*
	**Feeling informed**				1	−0.308**	−0.223**	−0.296**	−0.291**
**SOC**	**Total score**					1	0.883**	0.907**	0.773**
	**Comprehensibility**						1	0.690**	0.506**
	**Manageability**							1	0.612**
	**Meaningfulness**								1

Abbrev.: CTDQ: Confidence and Trust in Delivery Questionnaire; SOC: sense of coherence scale (29 items); VAS: visual analogue scale.

* = Significant (p < 0.05) ** = Highly significant (p < 0.01).

### External validity

To assess external validity, we correlated the CTDQ subscales with the SOC subscales (Table 3). We only observed some minor respective moderate correlations between r = −0.142 and r = −0.325 in absolute values. The strongest correlation was found between the SOC subscale “Manageability” with the CDTQ subscales “Confidence in labor” (r = −0.325) and Partner’s support (r = −0.321). Thus the percentages of shared variance indicated by the square of correlation coefficient r^2^ between the SOC and the CTDQ lies between 2% and 11% and indicates an independence of the scales.

Strong correlations however were recognized between the SOC subscales: all subscales were highly dependent with absolute correlation values ranging from r = 0.506 to r = 0.907. Thus, shared variance lies between 26% and 82% and underpins a interdependence of the SOC subscales. The highest correlations within the CDTQ was observed between the subscales “Trust in medical competence” and “Partner’s support (r = 0.702; r^2^ = 49%), while other subscales correlations ranged between r = 0,184 and 0,334 leading to a shared variance between 3% and 11%. A detailed overview is provided in Table 3.

### Subscales and parity

Scales both of the SOC and the CDTQ did not significantly differ with respect to parity status, age and educational level of the upcoming mothers. In all subgroup analyses score values showed a quite similar range and differences did not turn out to be significant between the groups (Table 4).

**Table 4:** SOC and CDTQ mean values subdivided for parity, age and educational level

	**Parity**		**Age**		**Education**		**Total**
	**Primipara**	**Multipara**	**≤ 32 years**	**>32 years**	**Lower**	**Higher**	
**SOC**							
Total score (Range 29–203)	150,7 ± 16,8	151,4 ± 17,5	151,8 ± 16,8	151,2 ± 18,2	151,9 ± 16,6	151,5 ± 17,5	151.5 ± 17.4
Comprehensibility (Range 11–77)	51,0 ± 7,5	50,5 ± 7,8	50,7 ± 7,7	50,9 ± 7,8	51,3 ± 8,6	50,8 ± 7,7	50.8 ± 7.7
Manageability (Range 10–70)	53,1 ± 7,3	53,5 ± 7,4	53,5 ± 7,1	53,6 ± 7,7	53,5 ± 6,4	53,6 ± 7,5	53.6 ± 7.4
Meaningfulness (Range 8–56)	46,6 ± 4,9	47,4 ± 4,8	47,5 ± 4,8	46,7 ± 5,3	47,1 ± 4,5	47,1 ± 5,1	47.1 ± 5.0
**CDTQ**							
Confidence in labor	3,1 ± 1,1	2,9 ± 1,1	2,9 ± 1,1	3,0 ± 1,1	3,1 ± 1,2	2,9 ± 1,1	3,0 ± 1,1
Partner’s support	1,6 ± 0,7	1,7 ± 0,7	1,7 ± 0,7	1,7 ± 0,8	1,7 ± 0,9	1,7 ± 0,7	1,7 ± 0,7
Trust in medical competence	1,8 ± 0,8	1,9 ± 0,9	1,9 ± 0,9	1,8 ± 0,9	1,9 ± 0,7	1,9 ± 0,9	1,9 ± 0,9
Feeling informed	2,5 ± 1,0	2,8 ± 1,2	2,7 ± 1,1	2,6 ± 1,1	2,9 ± 1,3	2,6 ± 1,1	2,7 ± 1,1

## Discussion

This paper aimed at evaluating the properties of the Confidence and Trust in Delivery Questionnaire (CTDQ) in 221 expectant mothers who attended the maternity ward of Havelhoehe Community Hospital for booking. Our study provided evidence of the reliability and criterion validity of the CTDQ.

The CTDQ was originally intended to establish a one-factor solution describing trust and confidence in labor that could apply in a global context; however, it turned out that the factor analysis arranged the items in a more discriminating way of 4 sufficiently independent factors. With its resulting subscales “confidence in labor”, “partner’s support”, “trust in medical competence” and “feeling informed”, we found dimensions which were sufficiently independent from Antonovsky’s SOC.

The results of this survey suggest that the core aspects of trust and confidence in labor are captured with this easy-to-use instrument comprising of only 11 items. Compared with the dimensions of other instruments and the findings of Hodnett’s review [5] from 2002, the instrument seems well-suited for use in research and evaluation.

Apart from the statistical and structural stability, the factors also have clinical implications as they are consistent with women’s own descriptions of what is relevant to them. Already in 1993, Bramadat suggested that perception of control during labor contributes to childbirth expectations [18]. In addition, Lally found that women prefer active involvement in labor management [19], thus making decisions about labor and having positive experiences also seems to dependent on “partner’s support” [20]. This was also recognized in the development of the Childbirth Expectations Questionnaire (CEQ), which includes this dimension like in our questionnaire. Taking these results into consideration, practical implications and relations like the high correlation of “partner’s support” with “trust in medical competence”, which we found in our study, should be investigated further.

The dimension “feeling informed” also seems to be of interest in other contexts. In a survey of 5.830 German health insurants that asked for their appraisal and use of complementary and alternative medicine (CAM), there was a significant correlation between searching for information and adaptive coping strategies in those participants using acupuncture and homeopathy, while trust in medical treatments was of significantly higher importance for nonusers [21]. According to a review by Adams et al. [22], there is a lack of “in-depth understanding of user experiences” and “comparing consumption patterns across cultures and over time” might be of interest in analyzing the score values of the CTDQ with respect to the usage of CAM-therapies in childbirth.

Due to organizational aspects, our experience with the CTDQ up to now is restricted to mothers with a more or less uncomplicated childbirth. However, for some women, birth is a traumatic event that significantly impacts their physical and emotional well-being [23]. Due to the compactness and easy usage of the CTDQ, it might be beneficial as a screening instrument, particularly with respect to the dimensions “confidence in labor” and “partner’s support” which seem to be relevant dimensions in the decision-making of mothers-to-be. In particular the instrument might be useful to explore how different types of antenatal care may influence the women’s confidence and trust in delivery. It might also be helpful in identifying upcoming mother’s need for counselling and support in the areas covered by the CTDQ.

Although not directly compared to related scales, we nevertheless had the SOC as external references. Our study did not offer any evidence that the SOC is an outcome measure of special interest for dimensions like trust and confidence as we found only moderate correlations of the CTDQ with the SOC. The results may be explained by the fact that there are some inconsistencies within the SOC that are not yet adequately examined [24] which were also present in our study by means of high internal correlations of the SOC subscales. Although we found a clear distinction of the CDTQ and the SOC scales, external validity of the CTDQ questionnaire is not sufficiently rolled out and should be focused in future studies.

### Limitations

The focus of this pilot study in its origins was clearly dominated by practical aspects like feasibility, patient compliance and manageability. We only used the SOC as external references and did not directly compare the CTDQ to related scales. Although the SOC scale seems to be a reliable, valid and cross-culturally applicable instrument, the lack of other questionnaires must nevertheless be regarded as a weakness of this pilot study as we cannot give any further empirical information on the relationship of the CTDQ with similar instruments like the CEQ and the W-DEQ, which at the time of the study were not available in a validated German version. Thus the results should be carefully interpreted. The current analysis cannot exclude that “confidence and trust in delivery” is not a unitary construct, but rather a set of items, each of which has its own independent meaning. Further studies with this instrument should provide clearer evidence for what the scale is measuring by comparing it with similar psychometric instruments.

From a psychometric perspective, it could also be argued that a suitable instrument should include more items, making the measurement of more aspects of labor possible. However, the main aim of this study was not to develop an instrument which captures all possible aspects of labor. On the other hand, the short format still allows for the opportunity to apply other instruments without a loss in compliance or a risk of overloading the expectant mothers. However, as 2 of the 4 subscales are only comprised of 2 items, an extension of the instrument in these dimensions with a subsequent re-analysis of its structure might be useful.

It is also noteworthy that our study results suffer from a lack of representativeness. Compared to other studies and the mean age of German mothers in 2008 (30.4 years) [25], the women in our cohort were slightly older (see Table 2). This might be due to the fact that our survey was not restricted to primipara women. Indeed, prior experience of labour has an impact on a women’s expectations of childbirth and therefore on the results on our confidence and trust in labour questionnaire. However our analysis did not find significant score differences in the SOC and CDTQ Scales between primipara and multipara.

Finally, women with higher education are known to be patients who seek integrative medical approaches. Thus, our results on the first sight seem not surprising considering the observed high percentage of expectant mothers with higher education. Surprisingly we failed to find significant differences in SOC and CDTQ-scores in women with higher education compared to those with lower educational level. This might be explained by the fact that Havelhoehe hospital apart from its integrative approach serves as a registered community hospital for routine patient care of that region.

## Conclusion

Apart from these limitations, the study presents a new and easy to fill out instrument available for expectant mothers. With 4 clinically relevant dimensions, a satisfactory internal consistency and first results on its external validity, the CTDQ is now open for further studies in the field of delivery care. We would like to see the CTDQ being used in other settings and with other instruments to improve its quality.

## Competing interests

The authors declare that they have no competing interests.

## Authors’ contributions

EJ, ND, DB and HM designed the study. ND collected the data. EJ and TO performed the statistical analysis and drafted the manuscript. All authors participated in the interpretation of the findings, reviewed the manuscript and approved the final manuscript.

## Pre-publication history

The pre-publication history for this paper can be accessed here:

http://www.biomedcentral.com/1472-6874/12/26/prepub

## Supplementary Material

Additional file 1 CTDQ-Original Items. Click here for file
