# Supplementary material for: Psychometric properties of the Confidence and Trust in Delivery Questionnaire (CTDQ): a pilot study
Source: BMC Womens Health. 2012 Sep 1;12:26. doi: 10.1186/1472-6874-12-26 (PMC3484078; doi:10.1186/1472-6874-12-26)
Supplement: Additional file 1 — CTDQ-Original Items. [file 1472-6874-12-26-S1.doc]

**CTDQ-Original Items**

**1) Die bevorstehende Geburt ist in erster Linie eine Quelle**

tiefer Freude 1 | 2 | 3 | 4 | 5 | 6 | 7 großer Sorgen und Ängste

**2) Wenn Sie an die bevorstehende Geburt und an die Geburtsschmerzen denken, neigen ich eher dazu**

es freudig zu erwarten 1 | 2 | 3 | 4 | 5 | 6 | 7 zu verzagen

**3) Alle Berichte und Erzählungen, die ich von Geburten kennen, haben mich insgesamt**

in meiner Freude und verunsichert und meine

Zuversicht gestärkt 1 | 2 | 3 | 4 | 5 | 6 | 7 Sorgen vermehrt

**4) Wenn ich an die Schmerzen denken, mit denen ich wahrscheinlich während der Geburt konfrontiert werden, haben ich das Gefühl, dass**

es mir gelingen wird, es mir nicht gelingen wird,

mit den Schmerzen zu mit den Schmerzen zu

Recht zu kommen 1 | 2 | 3 | 4 | 5 | 6 | 7 Recht zu kommen

**5) Dass mein Partner bei der Geburt dabei ist, ist mir**

sehr wichtig 1 | 2 | 3 | 4 | 5 | 6 | 7 unwichtig

**6) Während der Geburt wird es immer eine Person geben, auf die ich mich verlassen kann**

ganz sicher 1 | 2 | 3 | 4 | 5 | 6 | 7 sehr unsicher

**7-9) Auf die Unterstützung der folgenden Personen bei der Geburt kann ich vertrauen:**

Ganz und gar auf die ganz und gar nicht auf die

Hilfe des Partners 1 | 2 | 3 | 4 | 5 | 6 | 7 Hilfe des Partners

Hilfe der Ärzte 1 | 2 | 3 | 4 | 5 | 6 | 7 Hilfe der Ärzte

Hilfe der Hebamme 1 | 2 | 3 | 4 | 5 | 6 | 7 Hilfe der Hebamme

**10) Über die Möglichkeit einer PDA fühle ich mich informiert:**

Sehr gut 1 | 2 | 3 | 4 | 5 | 6 | 7 ungenügend

**11) Ich habe das Gefühl, auf die Geburt vorbereitet zu sein:**

Sehr gut 1 | 2 | 3 | 4 | 5 | 6 | 7 ungenügend
